# Macrophage Boolean networks in the time of SARS-CoV-2

**DOI:** 10.3389/fimmu.2022.997434

**Published:** 2022-10-17

**Authors:** Ugo Avila-Ponce de León, Osbaldo Resendis-Antonio

**Affiliations:** ^1^ Programa de Doctorado en Ciencias Biológicas, Universidad Nacional Autónoma de México, Ciudad de México, Mexico; ^2^ Human Systems Biology Laboratory, Instituto Nacional de Medicina Genómica (INMEGEN), Ciudad de México, Mexico; ^3^ Coordinación de la Investigación Científica – Red de Apoyo a la Investigación, Universidad Nacional Autónoma de México (UNAM), Ciudad de México, Mexico

**Keywords:** macrophage, COVID-19, Boolean models, systems immunology, gene regulatory network, SARS-CoV-2

## Introduction

The post-pandemic period of the current coronavirus disease (COVID-19), caused by severe acute respiratory syndrome coronavirus 2 (SARS-CoV-2), has lasted longer than expected despite the huge impact of the world-wide vaccination campaign in the past years (1). Since the pandemic began, endless mathematical models have been published to describe the viral outbreak at a population level. However, the molecular mechanism that drives the pathogenesis of the virus in the human microenvironment has been scarce (2–6). The existing mechanistic models deal to answer how SARS-CoV-2 invades the lung microenvironment and shapes the composition of the immune system in favor of virus duplication. From the clinical point of view, almost all patients that develop severe COVID-19 lead from a life-threatening acute respiratory distress syndrome (ARDS), which is associated with a hyper-inflammatory microenvironment and injury in the alveolar and lung epithelium (7). One of the cells associated with this syndrome is an uncontrolled hyper-activated macrophage-associated syndrome (MAS), which promotes a systemic inflammatory response that exacerbates the progress of the cytokine storm in the host (8, 9). Furthermore, single-cell high-throughput technologies applied in COVID-19 infections have shown that macrophages and monocytes are more abundant over other immune cells in bronchoalveolar lavage fluid (BALF) and peripheral blood mononuclear cells (PBMC) (10–13). In addition, the cytokines produced by monocytes and macrophages modulate the response of the immune system in patients with COVID-19 (14–16). For instance, there is evidence supporting that monocytes in severe patients have a lower expression of the human leukocyte antigen HLA-DRB1, which represses the activation of the immune response through the low production of foreign peptides (17–19). Besides, monocytes have a lower expression of interferon-stimulated genes in severe COVID-19 patients, resulting in the delay of the interferon response against SARS-CoV-2 (19). Furthermore, macrophages in severe COVID-19 patients are associated with overexpression of pro-inflammatory genes when compared with moderate COVID-19 patients (20). Altogether, these and other findings highlight the remarkable role that monocytes and macrophage polarization have in the progression of the disease.

Given the overwhelming complexity underlying the macrophage polarization during the SARS-CoV-2 infection, there is a need to develop computational/mathematical models to understand their regulatory principles and built testable hypotheses to lessen their lethal effect on human health. To this end, it is desirable that these mathematical models integrate transcriptional regulatory information and include the lung microenvironment for moving towards a global outlook of how SARS-CoV-2 and macrophages interact to favor the progression of the disease.

## A glance at COVID-19 mathematical models

While most of the mathematical models have been focused on the direction of epidemiology studies, some of them have been suggested to understand how the immune system responds to the lung when infected by SARS-CoV-2. Sadria et al. developed a system of ordinary differential equations (ODEs) that includes the two types of immune response: innate and adaptive. In this paper, the authors highlighted the relevance of the adaptive response through the mechanistic action of effector T and plasma cells, both considered the main factor to trigger the infection in the lung. Through their mathematical model, they explore the effect of three potential therapies and found that these have effective outcomes in the patient when applied in the early stages of the disease (21). In order to analyze more realistic models, Vaidya et al. (22) developed a mathematical model including parameters of infection in infected ferrets. To simplify the analysis, they incorporated one variable for the whole immune response as a soluble mediator of interferon type I, which has the ability to activate an antiviral response in macrophages (23). Given that the dynamics of infection of a ferret are shorter than humans and develop the same dynamics as the virus, this has been an interesting experimental model to explore how the virus infects host cells. Moreover, Li et al. developed a detailed multicompartmental mathematical model that integrated cytokine and immune cells in the peripheral blood and the trafficking to the lung. They focused on the activation of naive T/B cells by the antigen-presenting cells (APC) systems. The model predicted that a delay and an ineffective activation of T/B cells is associated with an increase in the viral load and that the constant absence of T/B cells is driven by myeloid-derived suppressor cells in severe patients of COVID-19 (24). Another compartmental model, calibrated with the viral charge of rhesus macaque infected with SARS-CoV-2, simulated the anti-inflammatory and pro-inflammatory immune response and concluded that the spread of the SARS-CoV-2 virus can be controlled by enhancing an innate response with an antiviral drug (25). Chowdhury et al. constructed a model that incorporated the interaction between the natural killer (NK) and T cells and explored how these cells had the ability to eliminate the virus by the influx of NK cells. Despite NK cells are not the most abundant cell in the lung, this study points out the importance of a fast and precise response of the NK cells of the innate immune system given their ability to secrete mediators that neutralize virus division (26). From a systemic perspective, a multiscale model was pursued by Wang et al, who incorporated the action of the cytokine storm and effector T cells (6). This data-driven study demonstrated that interferon can prolong the incubation period of the virus in the absence of T cells when patients transit from mild to severe symptoms. Voutouri et al. assimilated the importance of neutrophils and the secretion of their extracellular traps (NET) and evaluated how they shape the dynamics of the adaptive immune response. Currently, most of these mathematical models suggest that a delay in T cell function is associated with severe symptoms in the patients (27). For instance, Voutouri et al. supplied evidence of the relevant role that T cells have in the hyper-activation of the immune response and the cytokine storm (27). Other mathematical models have focused on the action of an antiviral response over the immune system, establishing the bases for theoretical therapies (28). Overall, these papers highlight directly or indirectly that two variables are important in the development of COVID-19: the microenvironment (cytokine storm, fibrosis mediators) and the cells conforming the immune system. In agreement with this fact, some mathematical models have evaluated the dynamics of cytokines and antigens in severe COVID-19 patients with fine levels of regulatory details. For instance, Reis et al. (29) developed a mathematical model of a set of fifteen ODEs comprising T and B cells, antibodies, and cytokines. Besides, the action of APC cells was included to mimic the innate immune response. With this model, they demonstrated that an environment with high cytokine secretion favors the virus and can infect immune cells. Other mathematical models have focused on analyzing the dynamical origin of the cytokine storm in severe COVID-19 patients. Sasidharakurup et al. developed a system of ODEs that incorporated cytokines and explored how their dysregulation affects the insulin production, thrombosis and nitric oxide pathways (30). In their simulation, they found that the most abundant cytokines, like IL1-*β*, IL-6 and tumor necrosis factor (TNF-*α*), can imbalance the homeostasis of the body. As well, they concluded that cytokines had the ability to maintain a positive feedback with other immune cells, much of it deriving in an uncontrolled cytokine storm (30). On the other hand, most of these models confirm that the nuclear-factor B(NFκB) is a crucial component that modulates not only the immune cells but also the inflammatory mediators. Notably, this transcription factor (TF) is activated in macrophages which is associated with an pro-inflammatory phenotype (31, 32). In reference (33) the authors developed a compartmentalized system of ODEs by which they assessed how detrimental is cytokine storm for healthy cells. In this last work, they simplified a single equation of all the possible cytokines that can be found in a COVID-19 inflamed lung. As a result, they concluded that the cytokine storm was the variable to focus with the therapies because it caused a decrease of healthy cells which made the recovery in severe patients harder (33). Furthermore, in reference (34) the authors developed a two-step system of ODEs whose variables represented the immune-cytokine and the standard SIV (susceptible-infected-virus) systems. Interestingly, they called attention to the fact that the cytokine storm may or may not be subject to a challenge of the immune system. In other words, they suggest some rare cases of individuals who develop a chronic infection without a storm-like immune-cytokine dynamics. This mathematical result suggests that the cytokine storm could be an individual-specific response to SARS-CoV-2, probably associated to factors such as the personalized genetic, sex, age and comorbidities in the patient. Undoubtedly, this last point requires more studies to be verified in clinical stages, a field that should be addressed in the next future (34). Altogether, this strongly suggest that fails in the immune system are responsible, directly or indirectly, for fomenting an augmentation in the cytokine storm. Despite monocytes and macrophages are the most abundant cell in an infected lung cell, to our knowledge there is no mathematical model that incorporates the transcriptional regulatory mechanism of macrophages for elucidating the dynamics in the progression of the disease.

## SARS-CoV-2 pathogenesis and its association with macrophage polarization

At present, the linkage between SARS-CoV-2 and the macrophage polarization can be summarized as follows. Macrophages sense the E protein of SARS-CoV-2 viral particles by using Toll-like receptor 2 (TLR2). Consequently, the activation of this receptor trigger downstream the mobilization of pro-inflammatory transcriptional factors that induce the secretion of IL-6, IL-8, and TNF-*α* among others signals involved in the cytokine storm (35). In addition, other toll-like receptors (1,4,5,8 and 9) are highly expressed in severe COVID-19 patients which activate pro-inflammatory cytokines (36). Interestingly, toll-like receptor 7 (TLR7), essential to trigger an antiviral response through the secretion of type I IFN-*α*/*β*, increases in moderate COVID-19 patients. Meanwhile, in severe COVID-19 patients, TLR7 decreases its expression because SARS-CoV-2 has the ability to inhibit pathways associated with the secretion of IFN-*α*/*β* (37).

Macrophages not only produce pro-inflammatory cytokines, but they also can be a target for the virus. Once invaded by the virus, macrophages secretes IL-6, TNF-*α*, IL-10, and cytokines that regulate the continuous activation of macrophages. The virus itself has developed structural and non-structural proteins to strictly inhibit the signaling towards the secretion of interferons. For example, open reading frame 3a (ORF3a) has the capacity to prevent the phosphorylation of STAT1, a TF associated with an interferon-based macrophage (38, 39). In addition, the M protein is associated with inhibiting the pathway of IFN-*α*/*β* and NFB, both pathways associated with the secretion of antiviral cytokines (40).

SARS-CoV-2 is not only the one factor that can trigger a hyper-inflammatory phenotype in macrophages, but also neutrophils and CD4+ T cells can stimulate the same response. Pro-inflammatory macrophage secretes IL-8, a potent chemoattractant for neutrophils (41, 42). Once in the lungs, they secrete extracellular traps (NET) to prevent pathogens from escaping the immune response, and the presence of an excess of NET is implicated in a poor outcome in COVID-19 (43). These NETs generate positive feedback between the cross-talk signals coming from macrophages and neutrophils, specifically NETs enhance the secretion of TGF-*β* (11, 44) and IL-1*β*, which further recruits more neutrophils in the lungs. Due SARS-CoV-2 infection is associated with the secretion of reactive oxygen species, neutrophils tend to die and be consumed by inflammatory macrophages, secreting TGF-*β* and IL-1*β* and creating a vicious cycle in favor of a pro-inflamatory environment (7). The secretion of TGF-*β* polarizes macrophages to an M2 state associated with the secretion of pro-fibrotic mediators which is implicated with fibrosis in the lungs (11, 13, 44). On the other hand, CD4+ TGehan Umali Cortezcells secrete the granulocyte colony-stimulating factor (GMCSF) an activator of a pro-inflammatory macrophage (45), and the hyper-activation of macrophages is associated with the depletion of CD8+ T lymphocytes, which is correlated with disease severity (46, 47). Altogether, these mechanisms conform to a complex environment by which the virus modulates the phenotype of the macrophage toward a pro-inflammatory stage, a situation with lethal consequences in the human host.

## Future directions for macrophages targeting agents for reducing the severity of COVID-19

In our opinion, two factors will contribute to unveil the genetic mechanism that guides the response of macrophages facing the SARS-CoV-2 infection: the high-throughput technologies and the computational/mathematical model of regulatory networks. On one hand, the importance of macrophages in COVID-19 has been evidenced thanks to the transcriptional profiles of thousands of single cells obtained from bronchoalveolar samples in COVID-19-infected patients (7, 20). Currently, these massive amounts of data can integrate the gene expression of thousands of genes in thousands of cells from a sample in time and space simultaneously (48). At this stage, the analysis of these data through machine learning (ML) algorithms can identify how the abundance of macrophages and other immunological cells could be associated with the progression and outcome of the disease. For example, by combining ML algorithms and single-cell RNASeq data from bronchoalveolar samples, we classified COVID-19 patients with different degrees of severity and found that genes associated with a pro-inflammatory response are implicated in severe COVID-19 patients (20). Besides, it has been reported that unsupervised hierarchical clustering is a proper method to stratify and classify patients through their severity progression determined by the pro-inflammatory, anti-inflammatory, and antiviral cytokines abundance data (49). By using this approach, the authors moved toward pragmatism and categorized an individual on its hospital admittance into high or low-risk categories (49). Simultaneously, other studies have been able to track immune cell subsets using an unsupervised algorithm (50). Another contribution of machine learning method in COVID-19 is that it enables us to track possible trajectories of differentiation from macrophages to T cells as the disease evolves. Specifically, these studies have concluded that the macrophage phenotypes changed with respect to disease severity, while T cells-related phenotypes did not. Macrophage phenotypes tilted to a more pro-inflammatory phenotype as severity increased (51). Finally, but not less important, some ML approaches have been used to differentiate flow cytometry profiles of blood samples from positive COVID-19 patients with respect to other diseases (like pneumonia) (52). On the other hand, the second factor that completes the global outlook is given by advanced techniques to gather biological data, and use them to build high-curated gene regulatory networks (GRN) in macrophages (32, 53). Finally, the computational modeling of these GRN constitutes an appealing framework to delimit the structure of the epigenetic landscape, this last a concept defined by Waddington in the mid-twentieth century (54). Notably, this last avenue was started some years ago with remarkable contributions in cancer to quantify the possible phenotype space for macrophages and its functional characterization in cancerous environments (32, 55). With the purpose to estimate the different phenotypes obtained from a GRN, a common approach is Boolean modeling. To simplify the complexity of the possible phenotypes emerging from the number of genes and their interactions in a GRN, this mathematical approach simplifies the description by considering two assumptions. First, one assumes that each gene can be in one of two feasible states: 0 (OFF) or 1 (ON). While the OFF state means that a transcription factor or gene is inactive, ON indicates that the gene is active. Second, we determine the state of each gene in the network through a Boolean function, which mathematically is represented by the combination of the logical operators AND, OR, and NOT. As far as possible, these logical functions are constructed from the experimental in order to improve the phenotypic landscape obtained from the GRN in a study (56, 57). Despite the simplicity, the Boolean network approach can allow us to tackle a set of relevant questions in immunological cells ranging from how *in silico* perturbation influences specific phenotypes, to the identification of those variables with the highest relevance in the secretion of cytokines, and those that could trigger the cytokine storm. In a pragmatical sense, the Boolean network approach generates testable hypotheses to evaluate the role that macrophages have in the hyper-inflammatory process during a SARS-CoV-2 infection. Thus, this formalism establishes the bases to tackle a set of questions that comprise the macrophage ability to transit among distinct phenotypes to the design of optimized strategies for preventing the committed activation of macrophages during COVID-19. For example, if we understand how the cytokines, chemokines, and viral products in patients with COVID-19 affect the transcriptional polarization of macrophages, we can try to tilt the balance of the hyper-inflammatory scenario to a more hyper/regulatory inflammatory by perturbing specific TF. The implementation of this formalism can drive strategies to limit a hyper-inflammatory stage in macrophages and avoid cytokine storm, lymphopenia, thrombosis, or other complications related to COVID-19 (58). Finally, more than ever the development of mathematical and computational strategies that help us understand the molecular mechanism of COVID-19 at a systemic level is a required activity with invaluable benefits at a long time scale to move from associations to mechanistic explanations, both areas needed to overcome the pandemic situation that we face around the world.

## Discussion and conclusion

Understanding the physiological response that emerged from the complex interaction between macrophages and SARS-CoV-2, is a valuable aim to potentially design effective treatments against COVID-19. Given the complexity of this pandemic disease, Boolean network modeling is one of the feasible strategies for a better understanding of how the disease starts and progresses. In this article opinion, we have focused on the role that macrophages play in COVID-19 given predominant relative abundance onto other cells of the immune system. To this end, we need to construct a highly curated signaling network between SARS-CoV-2-derived factors and macrophages. Interestingly, previous GRN of macrophages can help toward this purpose (32, 53, 55). Such a regulatory network should be able to condense well-known physiological knowledge of the macrophages and the information accumulated in the last years of the pandemic outbreak. We know that macrophages can be into two phenotypes: M1 and M2. M1 is associated with a pro-inflammatory phenotype while M2 is implicated with an anti-inflammatory phenotype. In an inflamed lung with SARS-CoV-2, there is an excessive amount of pro-inflammatory cytokines because of the cytokine storm. Pro-inflammatory macrophages (M1) are activated by TLR2 (which is activated by SARS-CoV-2) or by inflammatory cytokines like IL-6, IL1, TNF-*α* and IL-8 creating a positive feedback loop between the IL-6, IL1, TNF-*α* and IL-8 coming from the environment and those signals coming from the M1 macrophages. The TF responsible of the secretion of pro-inflammatory cytokines in macrophages is NFB. Another TF associated with M1 macrophage is STAT1 implicated in secreting IFN-γ and IFN-*β*, both molecules associated with viral clearance of SARS-CoV-2 (59–61). STAT1 decreases its expression (if not stimulated with IFN-γ), thus NFκB, in the absence of interferon gamma (IFN-) seems to maintain the M1-polarized system with liberation of cytokines to enhance the cytokine storm. M2a macrophage is activated by IL-4 or IL-13, nevertheless IL-4 was higher in severity than mild COVID-19 (62, 63). Despite having a greater amount of IL-4 the number of M2 macrophages were lower in COVID19 than H1N1 patients, which means that most of the IL-4 is tilted to a TH2 response associated with lymphopenia (64). This being said, we need more macrophage polarization to an M2a type because it is associated with fibrogenic inflammatory remodeling, promoting the secretion of TGF-*β* (which did not present any statistical difference between the comparisons (64)) allowing the formation of a temporary matrix and the proliferation of type II pneumocytes. M2c is a macrophage activated by IL-10, which is associated with the secretion of IL-10 and TGF-*β*. However, recent evidence shows that if IL-10 is secreted at the early process of inflammation it may have the ability to induce a pro-inflammatory action instead of an anti-inflammatory behavior. Because it is in a hyperinflammatory state due to the cytokine storm, IL-10 tries to temper and prevent tissue damage, but fails to do so which has been reported in arthritis (65, 66). Another possibility of the fail of IL-10 is the presence of high glucose in the blood (associated with diabetes), studies have shown that macrophages cultivated in high glucose uptake are implicated in an IL-10 resistance implicating in the inability to inhibit the secretion of pro-inflammatory cytokines (67). This means that IL-10 has to be perfectly secreted in the precise time to avoid in the inability to inhibit a pro-inflammatory response, so we can diminish the concentration of IL-10 by inhibiting the activation of M2c macrophages, and more macrophages associated with tissue repair. In conclusion, understanding how macrophages are regulated in a COVID-19 microenvironment could lead us to improve strategies to face this and future outbreaks. At present, the mathematical and computational models are invaluable schemes not only for understanding the molecular mechanism by which the SARS-CoV-2 evolves and transmit along the population, but also suggest strategies that in combination with health authorities help to decide the best actions for the benefit at local and global scales in the human population.

## Author contributions

All authors listed have made a substantial, direct, and intellectual contribution to the work and approved it for publication.

## Funding

The authors thank the financial support from PAPIIT-UNAM(IA202720). UA-P received a doctoral fellowship from Consejo Nacional de Ciencia y Tecnologıía (CONACYT) (CVU: 774988).

## Acknowlegdments

UA-P is a doctoral student from Programa de Doctorado en Ciencias Bioloígicas of the Universidad Nacional Autoínoma de Meíxico (UNAM). This paper was written during his PhD studies.

## Conflict of interest

The authors declare that the research was conducted in the absence of any commercial or financial relationships that could be construed as a potential conflict of interest.

## Publisher’s note

All claims expressed in this article are solely those of the authors and do not necessarily represent those of their affiliated organizations, or those of the publisher, the editors and the reviewers. Any product that may be evaluated in this article, or claim that may be made by its manufacturer, is not guaranteed or endorsed by the publisher.
